# Is There a Role of Targeted Agents in the Management of Adrenocortical Cancers?

**DOI:** 10.1155/2012/875764

**Published:** 2012-11-08

**Authors:** M. W. Saif, B. Fallon, K. N. Syrigos

**Affiliations:** ^1^Division of Hematology/Oncology, Tufts Medical Center, Tufts University School of Medicine, MA, USA; ^2^The Hospital of Central Connecticut, New Britain, CT 06489, USA; ^3^Oncology Unit GPP, Sotiria General Hospital, Athens School of Medicine, Mesogeion 152, 115 27 Athens, Greece

## Abstract

*Background*. Adrenocortical carcinoma (ACC) is a rare and aggressive tumor arising from the adrenal cortex with an incidence of one to two cases per million within the general US population. Recent developments in the understanding of the pathogenesis of ACC have led to multiple clinical trials involving targeted agents in the management of ACC. *Patients and Methods*. We report two cases of refractory adrenocortical cancer (cisplatin, adriamycin, etoposide, and mitotane) who were treated with targeted agents such as erlotinib and sutent, respectively. A total of 2 women with adrenocortical cancer were reviewed and followed for a median time of 6 months. Radiological response, duration of response and toxicities were evaluated. *Results*. In both cases, the targeted agents were able to control the disease for a short duration, but due to the deterioration in performance status and fatigue the agents were discontinued. *Conclusion*. The current observations emphasize the need for better targeted treatment modalities and strategies for the management of this fatal disease.

## 1. Introduction

Adrenocortical carcinoma (ACC) is a rare aggressive tumor arising from the adrenal cortex with an incidence of one to two cases per million within the general US population. The median age at diagnosis is 44 years and it is diagnosed at an advanced stage seventy percent of the time [1]. The only curative treatment in patients with early stage disease is radical surgical excision. Even after radical surgery, the recurrence rate is as high as 75 to 80 percent [2, 3]. The most significant prognostic factor is the tumor stage at diagnosis. Until recently, only few chemotherapeutic agents have shown to be beneficial in patients presenting with metastatic ACC. The uses of those agents were limited by their profound toxicity. Recent developments in the understanding of the pathogenesis of ACC have led to multiple clinical trials involving targeted agents in the management of ACC.

The management of adrenocortical cancer depends on the stage at which the cancer is diagnosed. Stages I and II which are confined to the adrenal glands are managed with radical surgery to remove the involved gland. Neither chemotherapy nor radiotherapy has not been shown to be beneficial in early stages. Patients who present with stage III disease are treated with surgery with or without regional lymph node dissection followed by chemotherapy. Patients with stage III disease, who are poor candidates for surgery or with local but unresectable tumor, can be managed with radiation (4200–5000 cGy for 4 weeks) to the site or with oral Mitotane. Palliative chemotherapy with mitotane/cisplatin alone is indicated in patients with stage IV disease, and local irradiation can be given in patients with bone metastasis for palliation. Clinical trials involving targeted therapies are underway.

It is well known that adrenocortical cancer is resistant to standard chemotherapy. The cause of this resistance has been attributed to the presence of high levels of a protein called MDR1 (multidrug resistance protein or P-glycoprotein) encoded by the ABCB1 gene. This membrane glycoprotein acts as a drug efflux pump, which pumps out chemotherapeutic agents such as vinblastine, paclitaxel, and doxorubicin [4]. Normal cells as well as cancerous adrenal cortical cells have high levels of MDR1 protein. The success of Mitotane in treatment of ACC is due in part to its ability to inhibit the MDR1 protein. Clinical trials with other MDR1 inhibitor compounds combined with standard chemotherapeutic agents have shown only modest benefits (Table 1). This indicates that there may be other MDR1-independent drug-resistance mechanisms which are responsible for the resistance. 

## 2. Patients and Methods

We discuss here, a case series of two patients who were treated with targeted agents for the diagnosis of adrenocortical cancer.

### 2.1. Patient Number 1

A 48-year-old female with past medical history of hypertension and uterine fibroids was being evaluated for a bump on the left side of her head. The lesion was resected in January 2007, which showed malignant epitheloid cells which was shown to be adrenocortical cancer. On immunochemistry Subsequent imaging revealed a large right kidney/adrenal mass with extension to the liver and nodules in the lung. CT head imaging was negative for metastatic disease. The scalp lesion recurred in March 2007 and was resected again with negative margins. Patient was evaluated by a Urologist for possible resection of mass, which was deferred due to size and extension of the mass. Patient was subsequently started on chemotherapy with cisplatin, adriamycin, VP-16, and mitotane. After the completion of three 4-week cycles of the above regimen, patient was started on mitotane daily. A CT chest/abdomen/pelvis was done 3 months after the first two cycles, which showed tumor progression. Patient completed the 4 cycles of chemotherapy, and restaging was done with a repeat CT chest abdomen and pelvis. Patient was started on Sutent, after 2 cycles as the disease was progressing; Sutent was changed to Tarceva in January 2008. Patient was scheduled to start on CPT11/docetaxel in March 2008. In the meantime patient's performance status deteriorated and chemotherapy was discontinued, and subsequently patient was referred for hospice care.

### 2.2. Patient Number 2

A 59-year-old female was being evaluated for left lingular and lower lung nodule in July 1999. Left thoracotomy was performed, which reveled metastasis from adrenocortical cancer. As the patient was asymptomatic at that time, no chemotherapy was given. In 2002 patient was found to have right-middle lobe nodule and left-lower lobe lesions. Patient had again a left thoracotomy in September 2004 for left-lower lobe resection, which showed carcinoid tumor. Patient was found to have scalp lesions in April 2006 and May 2008, which turned out to be metastatic adrenocortical cancer. Patient was started on mitotane in November 2008. In January 2009, patient was found to have a new scalp lesion for which she received radiotherapy for 2 months. As the patient was progressing, mitotane was discontinued and the  patient was started on cisplatin and etoposide. Patient was enrolled in a clinical trial with MK 5108 with docetaxel on May 2009, which was continued until February 2010. As the disease progressed, the regimen was changed to Sutent from March 2010 to July 2010.

## 3. Results

The first patient tolerated erlotinib (tarceva) for more than four months, but the drug was discontinued due to the deterioration in the patient's performance status as well as worsening lower extremity edema as a result of inferior venocaval invasion of the tumor. The second patient had stable disease on Sutent for 4.5 months after which the drug was stopped upon the patient's request due to fatigue.

## 4. Discussion

Adrenocortical cancers are aggressive neoplasms with dismal prognosis. Mitotane is the only FDA approved agent in the treatment of ACC, the use of which is often limited by its profound toxicity. Better understanding of the pathogenesis of these tumors has lead to the development of various targeted agents to treat these tumors. Here we are discussing some of the recent developments in the treatment of ACC. 

### 4.1. EGFR

Presence of EGFR in adrenocortical tumors has been demonstrated in the past [5]. The expression of EGFR in adrenal tumors indicates a malignant phenotype, which can be used to differentiate carcinomas from adenomas and is a potential target for treatment [6]. Although EGFR expression level did not correlate with the clinical outcome in patients, in vitro experiments demonstrated that inhibition of EGFR signaling lead to moderate growth inhibition in ACC cells [7].

### 4.2. VEGF

The level of VEGF was found to be high in patients with adrenal carcinoma compared to other adrenal tumors and [8] Moreover, there is a direct correlation between VEGF level and stage, size, and recurrence of the adrenocortical cancer [8, 9]. There are few ongoing trials, which evaluate the effectiveness of VEGF inhibitors in adrenocortical cancers [10–12]. 

### 4.3. IGF1/IGF 2/IGF-1R

Studies have shown that IGF related genes are over expressed in ACC tissues. IGFs elicit its cellular effects through the IGF receptors [13]. It has been demonstrated in the past that in human ACC samples, there is an over expression of IGF-1R and its ligand. Antagonizing the IGF signaling pathway has been shown to inhibit the Cell line growth of ACC cell lines in vitro and in vivo. These observations gave the possibility of using IGF-1R antagonists in treatment of patients with ACC in conjunction with Mitotane and other chemotherapeutic regimens. Clinical trials in this direction are underway [13]. 

### 4.4. mTOR

Mammalian target of rapamycin (mTOR) is an enzyme in the cellular phosphatidylinositol 3-kinase (PI3 K) pathway commonly associated in human cancer pathogenesis. New drugs have been developed to inhibit PI3 K/Akt signaling activation in cancer chemotherapy. In vitro and in animal models mTOR inhibitors (RAD001; everolimus) have shown to inhibit adrenocortical tumor cell proliferation [14] and [15]. These findings suggest mTOR antagonists as a potential treatment option in ACC patients (Table 2).

Better understanding of molecular pathogenesis of ACC should lead to the development of better therapies and effective drug designs. There is a need for world wide multicenter collaborative research protocols for improved treatments as the incidence of the disease is very low. The current observations emphasize the need for better targeted treatment modalities and strategies for the management of this fatal disease.

## Figures and Tables

**Table 1:** Current clinical trials for adrenocortical cancer (from http://www.cancer.gov).

Trials	Agents Used	Additional Details	Organization
Phase I			

Study of Sorafenib and Bevacizumab in patients with refractory, metastatic, or unresectable solid tumors	Sorafenib plus Bevacizumab	Determine biochemical changes in the Ras-Raf-MAPK, and VEGF signal transduction pathways in tumor and stromal cells from patients treated with this regimen.	*NCI—Center for Cancer Research* (NCT00098592)

IMC-A12 in combination with Temsirolimus (CCI-779) in patients with advanced cancers	combination of IMC-A12 and Temsirolimus	Researchers will also perform biomarker tests to study how IMC-A12 and Temsirolimus affect genes	*MD Anderson Cancer Center* (NCT00678769)

Phase II			

Study of combination comprising Tariquidar, Mitotane, Doxorubicin, Vincristine, and Etoposide and surgery in patients with recurrent, metastatic, or primary unresectable adrenocortical cancer	Tariquidar, Mitotane, Doxorubicin, Vincristine, and Etoposide	*Primary outcome*: response rate (partial or complete)progression-free survival	*NCI—Center for Cancer Research* (NCT00073996)

Trial with Taxotere and Cisplatin in nonoperable adrenocortical carcinoma	Cisplatin plus Taxotere	*The primary objective*: to investigate response rate. *Secondary endpoints* are survival, time to progression, best overall response rate, and duration of response.	*Rigshospitalet-Copenhagen University Hospital* (NCT00324012)

Randomized study of Mitotane with versus without anti-IGF-1R recombinant monoclonal antibody IMC-A12 in patients with recurrent, metastatic, or primary unresectable adrenocortical carcinoma	Mitotane with IMC-A12 versus Mitotane without IMC-A12	*Primary outcome*: progression-free survival, objective response rates, changes in tumor size over time, and overall survival	*University of Chicago Cancer Research Center* (NCT00778817)

Study of Cixutumumab in patients with relapsed or refractory solid tumors	Cixutumumab	*Primary outcome*: response rate toxicity	*Children's Oncology Group* (NCT00831844)

Study of R-(-)-gossypol acetic acid in patients with recurrent, metastatic, or primary unresectable adrenocortical carcinoma	R-(-)-gossypol acetic acid.	Proportion of patients who achieve a confirmed objective response (complete or partial response by RECIST criteria) to treatment	*Mayo Clinic Cancer Center* (NCT00848016)

Study of axitinib (AG-013736) with evaluation of the VEGF-pathway in metastatic, recurrent or primary unresectable adrenocortical cancer	Axitinib (AG-013736)	Determine the response rate of Axitinib (AG-013736) in recurrent, metastatic, or primary unresectable ACC	*National Cancer Institute* (NCT01255137)

Sorafenib plus Paclitaxel in adreno-cortical-cancer patients	Sorafenib plus weekly Paclitaxel	*Primary outcome*: progression-free survival rate ≥ 40% after 4 months. Response rate evaluation.	*University of Turin, Italy* (NCT00786110)

Phase III			

GALACCTIC: a study of OSI-906 in patients with locally advanced or metastatic adrenocortical carcinoma	OSI-906	A multicenter, randomized, double-blind, and placebo-controlled phase 3 study of single-agent OSI-906 in patients with locally advanced/metastatic ACC who received at least one but no more than two prior drug regimen	*OSI Pharmaceuticals Incorporated* (NCT00924989)

Study of neoadjuvant and adjuvant Cisplatin-based chemotherapy and/or surgical resection in young patients with stage I–IV adrenocortical tumor	Oral mitotane; cisplatin IV; Etoposide IV and doxorubicin hydrochloride IV		*Children's Oncology Group* (NCT00304070)

**Table 2:** The cell markers and inhibitors in clinical trials.

Marker	Inhibitors in clinical trials
IGF 1 and 2 IGF-1 receptor	IMC-A12 (Cixutumumab) OSI-906

Vascular endothelial growth factor (VEGF)	Sorafenib Axitinib Bevacizumab

mTOR (mammalian target of rapamycin)	Temsirolimus
